# Modelling α-Synuclein Aggregation and Neurodegeneration with Fibril Seeds in Primary Cultures of Mouse Dopaminergic Neurons

**DOI:** 10.3390/cells11101640

**Published:** 2022-05-13

**Authors:** Aurore Tourville, David Akbar, Olga Corti, Jochen H. M. Prehn, Ronald Melki, Stéphane Hunot, Patrick P. Michel

**Affiliations:** 1 Paris Brain Institute-ICM, Inserm, CNRS, Hôpital de la Pitié Salpêtrière, Sorbonne Université, 75013 Paris, France; aurore.tourville@icm-institute.org (A.T.); david.akbar@icm-institute.org (D.A.); olga.corti@icm-institute.org (O.C.); stephane.hunot@icm-institute.org (S.H.); 2 Department of Physiology & Medical Physics and FutureNeuro Centre, Royal College of Surgeons in Ireland, University of Medicine and Health Sciences, D02 YN77 Dublin, Ireland; jprehn@rcsi.ie; 3 MIRCen, CEA and Laboratory of Neurodegenerative Diseases, CNRS, Institut François Jacob, 92265 Fontenay-aux-Roses, France; ronald.melki@cnrs.fr

**Keywords:** α-Synuclein, cell culture model, dopamine neurons, fibril seeds, neurodegeneration, Parkinson disease, protein aggregation

## Abstract

To model α-Synuclein (αS) aggregation and neurodegeneration in Parkinson’s disease (PD), we established cultures of mouse midbrain dopamine (DA) neurons and chronically exposed them to fibrils 91 (F91) generated from recombinant human αS. We found that F91 have an exquisite propensity to seed the aggregation of endogenous αS in DA neurons when compared to other neurons in midbrain cultures. Until two weeks post-exposure, somal aggregation in DA neurons increased with F91 concentrations (0.01–0.75 μM) and the time elapsed since the initiation of seeding, with, however, no evidence of DA cell loss within this time interval. Neither toxin-induced mitochondrial deficits nor genetically induced loss of mitochondrial quality control mechanisms promoted F91-mediated αS aggregation or neurodegeneration under these conditions. Yet, a significant loss of DA neurons (~30%) was detectable three weeks after exposure to F91 (0.5 μM), i.e., at a time point where somal aggregation reached a plateau. This loss was preceded by early deficits in DA uptake. Unlike αS aggregation, the loss of DA neurons was prevented by treatment with GDNF, suggesting that αS aggregation in DA neurons may induce a form of cell death mimicking a state of trophic factor deprivation. Overall, our model system may be useful for exploring PD-related pathomechanisms and for testing molecules of therapeutic interest for this disorder.

## 1. Introduction

Parkinson’s disease (PD) is a common neurodegenerative disorder of aging primarily characterized by a loss of voluntary motor control. Motor control abnormalities result from the progressive loss of midbrain substantia nigra (SN) dopamine (DA) neurons and subsequent nigro-striatal dopaminergic deafferentation [1,2]. Another major histopathological hallmark of this disorder is the presence of intracellular proteinaceous inclusions called Lewy bodies (LBs) in neuronal somas and Lewy neurites (LNs) in neuronal processes [3]. LBs are considered a key marker of neuronal degeneration in PD since they are found at sites particularly predisposed to neuronal loss—the SN and the locus coeruleus [4].

LBs are complex intracellular inclusions made of lipid membrane fragments and distorted organelles combined with α-Synuclein (αS), a membrane protein made up of 140 amino acids whose physiological function is still not fully understood [3,5,6]—even if some have proposed that it may contribute to neurotransmitter exocytosis through the dilation of synaptic vesicle pores [7]. Apart from the fact that LBs are enriched in insoluble forms of αS, there are two other main reasons to believe that αS contributes crucially to PD pathophysiology: (i) point mutations, duplications or triplications of the αS gene *SNCA* cause inherited forms of PD with LBs [8,9]; (ii) Genome-wide association studies have identified common αS variants as genetic risk factors for PD [10].

αS monomers are dynamic and can interact with membranes of different lipid compositions [11]. Upon binding to membranes, αS adopts an α-helical conformation essential for its biological function. In a pathological context, αS monomers form high molecular weight β-sheet-rich structures [12] consisting of stable oligomers, which are potentially cytotoxic, and fibrils that can drive the templated aggregation of αS [13,14,15,16]. It has been proposed that fibrillary species of αS propagate from neuron-to-neuron and between interconnected brain regions in a prion-like manner, thus promoting the spatial and temporal spread of the synucleinopathy [17,18,19]. The diffusion capacity of fibrillary species across cell membranes, as well as their retrograde or anterograde transport along neurites, are likely to contribute to the spread of pathological αS [20,21].

Given the probable link between αS aggregation and dopaminergic cell loss in PD, a number of groups have established cell culture systems to study αS-mediated neurodegeneration. Some of these models include rat (PC12) or human (SH-SY5Y) cell lines [22,23,24], which can be converted into a dopaminergic phenotype by differentiation. Undoubtedly, these cell lines represent valuable tools for studying αS-mediated pathology, but they derive from cancer cells and may therefore not recapitulate all the physiological features of terminally differentiated DA neurons. Conditionally immortalized LUHMES cells, which can also acquire a human dopaminergic phenotype after a differentiation step, represent an alternative to conventional cell lines [25,26]. Yet, differentiated LUHMES cells appear to be difficult to maintain over long periods of time, which may be a limitation when studying pathological αS aggregation. Patient-derived iPS cell lines differentiated into DA neurons appear to currently be most relevant for modelling αS aggregation-related processes, but generating iPS-derived DA neurons is a time-consuming process that also requires a high degree of technical expertise [27,28,29]. Importantly, none of these model systems reproduces the phenotypic diversity of neuronal cell populations within the midbrain, which may be an issue when considering the differential vulnerability of DA neurons to PD neurodegeneration.

Hence, our main objective was to develop a model system of αS aggregation in DA neurons that would be pathophysiologically relevant and easy to replicate. Our strategy was to use midbrain mouse dopaminergic cultures treated with fibril seeds generated from recombinant human αS to stimulate the templated recruitment of endogenous αS into LB-like aggregates. Specifically, our aim was to utilize this culture model system (i) to characterize the efficiency and kinetics of the aggregation process within DA neurons; (ii) to determine whether mitochondrial deficits, another prominent causal factor for PD neurodegeneration [30,31], could exacerbate αS aggregation and its consequences; (iii) to define under what specific conditions αS aggregation results in the dysfunction and ultimately the death of DA neurons; and (iv) to determine to what extent αS aggregation and/or associated neurodegenerative events are preventable.

To our surprise, we found that seeded aggregation of αS occurred preferentially in DA neurons in midbrain cultures through a process that is not dependent on DA metabolism. Noticeably, the impact of αS seeded aggregation was not aggravated by toxin-induced mitochondrial deficits or genetically induced dysfunction of mitochondrial quality control mechanisms. Yet, we established that αS aggregation ultimately results in a loss of DA neurons that is preventable by trophic stimulation with GDNF.

## 2. Materials and Methods

### 2.1. Use of Animals

Animals were housed, handled, and cared for in strict accordance with the European Union Council Directives (2010/63/EU). Experimental protocols were approved by the Committee on the Ethics of Animal Experiments Charles Darwin n^o^ 5.

### 2.2. Midbrain Cell Culture Protocol

Midbrain cultures were generally prepared with gestational day 13.5 embryos from C57BL/6J mice (Janvier LABS; Le Genest St Isle, France). In some experimental protocols, we also used day 13.5 embryos from Swiss mice (Janvier LABS) or *Parkin* knockout (*PRKN^−/−^*) mice brought into the C57BL/6J genetic background as described previously [32]. Experimental groups of age-matched littermates of *PRKN^−/−^* and wild-type mice were generated in-house by intercrossing heterozygous *PRKN^+/−^* mice.

Mice carrying E13.5 embryos were euthanized by CO_2_ inhalation followed by decapitation. Midbrains from mouse embryos were then dissected under a stereomicroscope and collected on ice in a 15 mL sterile polypropylene conical tube containing 2 mL of Leibovitz L15 culture medium (Sigma Aldrich; L’lsle-d’Abeau Chesnes, France). Tissue pieces were mechanically dissociated by gentle pipetting (8–10 strokes) using a Gilson pipette fitted with a sterile polypropylene blue tip with no filter (StarLab France, Orsay, France). The pipette was set to 950 μL to avoid accidental reflux and contact at the open end of the pipette. L15 medium was then added to complete the total volume to 8 mL and cells in suspension were gently mixed twice by inversion of the test tube. Non-dissociated tissue and cellular debris were then allowed to settle down at 4 °C for 30 min and 6.5 mL of the resulting supernatant containing dissociated cells in suspension was transferred to another sterile polypropylene conical tube, while the remaining pellet was taken for another round of trituration. The final supernatants (2 × 6.5 mL) were then centrifuged at 1300 rpm for 5 min at 4 °C and cells from the resulting pellets gently resuspended in 2 mL L15 medium before plating.

### 2.3. Maintenance and Treatment of Midbrain Cultures

Dissociated cells in suspension were seeded at a density of 40–60 × 10^3^ cells/cm^2^ onto either Ibidi μ-slide 8 well glass-bottom (CliniSciences, Nanterre, France) or Nunc 48-well multiwell plates (Roskilde, Denmark) pre-coated with 1 mg/mL polyethylenimine (PEI; P3143; Sigma Aldrich, L’Isles d’Abeau Chesnes, France) dissolved in a pH = 8.3 borate buffer [33]. The coating of culture surfaces with PEI greatly facilitated the maintenance of the cultures and subsequently their processing for analysis as this polycationic polymer possesses optimum attachment properties for brain cells [34,35]. The cultures were initially maintained in Neurobasal-A medium (#10888022; Thermo Fisher Scientific, Courtaboeuf, France) supplemented with B27 supplement minus antioxidants (#10889038; Thermo Fisher Scientific), a N2 mix (#17502048; Thermo Fisher Scientific), a cocktail of penicillin/streptomycin and 1% fetal calf serum (FCS; Biowest LLC, Les Ulis, France). Two and eighteen hours after plating, Ara-C was added to the cultures at a concentration of 0.8 μM to halt glial cell proliferation [34]. The cumulative concentration of 1.6 μM was not toxic for neuronal cells. On day 4 in vitro (DIV), the plating medium was entirely removed and replaced by astrocyte-conditioned medium (ACM), prepared as described thereafter. Half of the ACM was then replaced twice a week to ensure the optimal maintenance of the cultures. The ACM not only facilitated culture–medium exchange but also promoted neuronal development as well as spontaneous network activity, thus mimicking as closely as possible brain physiological conditions [36]. Pharmacological treatments used to modulate αS seeded aggregation and its consequences were renewed at the time of ACM replacement. Under current conditions, astrocytes and microglial cells represented <3% of the total number of cells in the mature midbrain cultures.

### 2.4. Production of Astrocyte-Conditioned Medium

ACM was prepared from primary cultures of astrocytes obtained from the brain of newborn C57BL/6J mouse pups. Astrocytes were grown onto laminin (1 μg/mL in distilled water)-coated T75 Corning culture flasks containing 12 mL of Dulbecco’s Modified Eagle Medium/F-12 nutrient mixture (Thermo Fisher Scientific, Courtaboeuf, France) supplemented with 10% FCS, 1% of a penicillin/streptomycin cocktail and 3 μg/mL clodronate liposomes (Liposoma BV, Netherlands). These culture conditions were reported earlier to favor the isolation of astrocytes while eliminating microglial cells [37,38]. Ten to twelve days after plating, the isolation medium was totally removed. Then, the cultures were washed twice with Dulbecco’s phosphate buffered saline (PBS) before being incubated with Neurobasal-A medium containing the same supplements as before, except for the FCS. After three days of conditioning in astrocyte cultures, the medium was recovered and frozen at −20 °C until use. Astrocyte cultures were used to produce ACM over three weeks.

### 2.5. Immunodetection Protocols

After a single wash with PBS, cultures were fixed for 12 min at room temperature with formaldehyde (3.7%) diluted in PBS. After washing with PBS, cultures were incubated with primary antibodies for 24–48 h at 4 °C. All primary antibodies were diluted in PBS containing 0.2% Triton X-100. The primary antibodies used in this study are described with their working concentrations in Table 1. A list of secondary antibodies and their conditions of use are described in Table S1.

### 2.6. Production of αS Fibrils

To model pathogenic αS aggregation, we used fibrils 91 (F91) seeds generated and characterized based on procedures described previously [39,40]. To be precise, human wild-type αS expressed in E. coli BL21 DE3 CodonPlus cells was purified and dialyzed overnight at 4 °C against 1000 times the volume of 20 mM NaP04 and 150 mM KCl—pH 9.1. The protein concentration was adjusted to 300 μM and the solution was incubated at 37 °C under continuous shaking in an Eppendorf Thermomixer set at 600 r.p.m. for 7 days. The assembly reaction was monitored by thioflavin T binding using a Cary Eclipse Fluorescence Spectrophotometer (Varian Medical Systems Inc., Palo Alto, CA, USA) set to excitation and emission wavelengths of 440 nm and 480 nm, respectively. The nature of the fibrillar assemblies was assessed by transmission electron microscopy after adsorption of the fibrils onto carbon-coated 200 mesh grids and negative staining with 1% uranyl acetate. Images obtained with a Jeol 1400 transmission electron microscope were recorded via a Gatan Orius CCD camera (Gatan, Pleasanton). The resulting fibrils were centrifuged twice at 15,000× *g* for 10 min and re-suspended twice in PBS. Their concentration was adjusted to 350 μM in PBS. They were next fragmented to an average length of 40–50 nm by sonication for 20 min in 2 mL Eppendorf tubes using a Vial Tweeter powered by an ultrasonic processor UIS250 v (250 W, 2.4 kHz; Hielscher Ultrasonic). Fragmented fibrils were aliquoted (6 μL) in Eppendorf tubes, flash frozen in liquid nitrogen and stored at −80 °C until use. In some experiments, we also used F91 fluorescently labelled with ATTO-550 (^ATTO^F91). Fluorescent labelling was achieved with ATTO-550 NHS-ester by adding two molar equivalents of the extrinsic fluorophore in DMSO to unfragmented fibrils. After incubation at room temperature for 1 h, the fibrils were centrifuged twice at 15,000× *g* for 10 min and re-suspended twice in PBS, before being fragmented as described above. Note that treatments with F91 or ^ATTO^F91 were carried out only once regardless of the post-seeding lag period.

### 2.7. Manual Cell Counting Operations

Manual cell counting operations were carried out with a Nikon Eclipse Ti-U fluorescence inverted microscope (Nikon France, Champigny sur Marne, France) equipped with a Hamamatsu’s ORCA-D2 camera and HCImage software (Hamamatsu Photonics, Massy, France). The number of tyrosine hydroxylase immunopositive (TH^+^) neurons/culture well was estimated by visually inspecting samples with a 10× objective over 10–15 visual fields that were randomly selected for each treatment condition. The number of TH^+^ somas containing αS aggregates (αSa) was estimated by visual examination of 10 visual fields that contained at least one TH^+^ neuron but were otherwise randomly chosen. The total number of Microtubule-Associated Protein-2 (MAP-2^+^) neurons and the number of MAP-2^+^ somas with αSa were estimated using 10 microphotographs of midbrain cultures that were acquired randomly with a 20X objective.

### 2.8. Confocal Imaging and 3D Image Analysis

Confocal imaging was performed using a Nikon A1R HD25 microscope (Nikon France). Images from cultures grown on glass-bottom Ibidi μ-slides were acquired every 0.175 μm in the Z direction using a 60× oil immersion objective (TIRF 60XC Oil; NA 1.49). For cultures grown onto polystyrene Nunc 48-well multiwell plates, we used a 20× water immersion objective (Lambda 20×; NA 0.95) and a Z-stack step size of 0.575 μm. All acquisitions were performed under the resonant scanner mode.

Three-dimensional image reconstruction for illustrations was performed with the open source image processing package FIJI [41] or the NIS-Elements imaging software from Nikon. Illustrations are presented as 3D projections, Z-projections or single optical sections. Note that in some cases, Z-projections of fluorescent images were subjected to an invert function that converts blacks to whites and whites to blacks and inverts gray levels proportionally, to allow for the better visualization of the fine details of neuronal morphology [42]. The resulting images are referred to as “inverted images”.

The 3D image reconstruction analyses were performed using the general analysis 3D option in the NIS-Elements software (Nikon). The aggregation process in dopaminergic neurites was analyzed in a confocal volume defined by a vertical projection column (*z*-axis) of a 100 μm radius centered on individual isolated TH^+^ cell bodies.

### 2.9. DA Uptake Measurements

The functional integrity and synaptic function of DA neurons were evaluated by the ability of these neurons to accumulate [^3^H]-DA by active transport (50 nM; 40 Ci/mmol; PerkinElmer, Courtaboeuf, France), as previously described [43]. GBR-12909 (0.2 μM; Sigma Aldrich, L’Isles d’Abeau Chesnes, France) was used to obtain blanks that were subtracted from raw values.

### 2.10. Statistical Analysis

Data expressed as means ± SEM were analyzed using SigmaPlot 12.5 software (Systat Software Inc., San Jose, CA, USA). Each data point was derived from at least two independent sets of experiments. Data were assessed by one-way analysis of variance followed by post-hoc Dunnett’s test for all comparisons against a control group, or the Student–Newman–Keuls (SNK) post-hoc test for all pairwise comparisons.

## 3. Results

### 3.1. Detection of αS Aggregation in DA Neurons with Anti-αS (Phospho-Ser129) Antibodies

Phosphorylation of αS at the Ser129 residue has been associated with αS aggregation and LB formation [44,45]. Therefore, we wished to test the efficacy and specificity of different anti-αS (phospho-Ser129) antibodies to detect the formation of αSa in DA (TH^+^) neurons from cultures exposed to αS fibrillar seeds. Specifically, we used three-week midbrain cultures exposed two weeks earlier to 0.5 μM F91, i.e., experimental conditions leading to the formation of large inclusions of phosphorylated αS (p-αS) in DA cell somas. Immunodetection of p-αSa was carried out with either rabbit recombinant monoclonal antibody EP1536Y (ab51253; Abcam; Cambridge, UK) or mouse monoclonal antibodies referred as p-Syn#64 (W1W015-25191; Fujifilm Wako Chemicals, Osaka, Japan) and p-Syn#81A (ab184674; Abcam). Co-immunodetection of TH was performed with a chicken polyclonal antibody (ab76442; Abcam). See Table 1 and Table S1 for details concerning primary and secondary antibodies, respectively.

**Figure 1:**
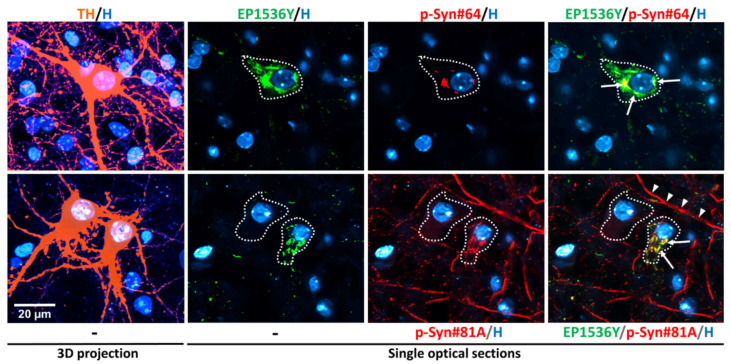
Comparative assessment of anti-αS (phospho-Ser129) antibodies for the detection of αSa in midbrain cultured DA neurons. The confocal images above are from three-week midbrain cultures previously exposed two weeks before to 0.5 μM F91. *Upper panel:* 3D projection of an individual TH^+^ (orange pseudocolor for deep-red fluorescence) neuron and a single optical section from the same neuron, revealing the presence of a large somal αS inclusion identified with EP1536Y (green) and p-Syn#64 (red) antibodies. The merged image shows that EP1536Y detects a much larger portion of the inclusion than p-Syn#64. *Lower panel:* 3D projection of two DA neurons (orange) and single optical section showing a large somal αS inclusion in one of the two DA neurons using EP1536Y (green) and p-Syn#81A (red) antibodies. The green and red immunosignals are well superimposed in the soma of the DA neuron with the inclusion, but the red label is also seen in elongated cellular elements that obviously do not contain aggregates. Nuclear counterstaining was performed with Hoechst-33342 (H; blue). White arrows depict coincident p-αS immunosignals (yellow) whereas white arrowheads point to non-specifically labelled structures.

Single optical sections from confocal images revealed that the three antibodies were effective in detecting p-αSa in individual DA cell bodies (Figure 1). We found that 90.0 ± 5.8% and 96.7 ± 3.3% of aggregates primarily identified with EP1536Y in DA cell bodies were also detectable with p-Syn#64 or p-Syn#81A, respectively (*n* = 25–29 neurons/group).

While p-Syn#64 detected only a small portion of each inclusion identified with EP1536Y, immunosignals for EP1536Y and p-Syn#81A overlapped relatively well in the somal compartment of DA neurons (Figure 1). Unlike EP1536Y, however, p-Syn#81A labelled elongated neuropil elements that were obviously not protein aggregates. Off-target staining was also detectable in control cultures not exposed to fibril seeds (not shown). Based on these elements, we selected the EP1536Y antibody for all subsequent experiments.

### 3.2. Seeded αS Aggregation in DA Neurons Increases Primarily with Fibril Concentrations and Post-Exposure Time to Fibrils

**Figure 2:**
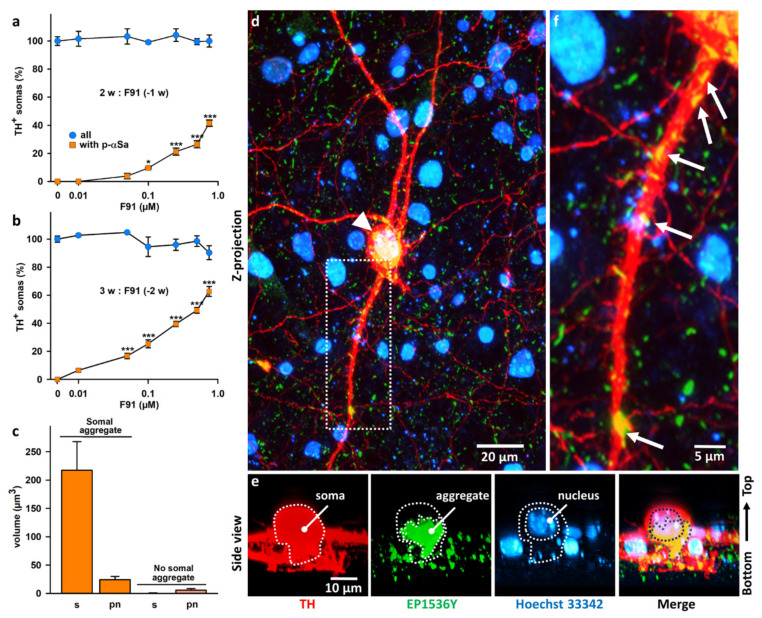
Quantitative and qualitative evaluation of αS seeded aggregation in midbrain cultured DA neurons. (**a**) Percentage of DA (TH^+^) cell somas with p-αSa and survival of these neurons in two-week midbrain cultures exposed one week earlier (−1 w) to increasing concentrations of F91 (0.01–0.75 μM = 0.14–10.7 μg/mL). (**b**) Same parameters as in (**a**) measured in three-week midbrain cultures exposed two week earlier (−2 w) to the same concentrations of F91. Data in (**a**,**b**) are means ± SEM (*n* = 3–10). * *p* < 0.05; *** *p* < 0.001 vs. controls. One-way ANOVA followed by Dunnett’s test. (**c**) Average volume occupied by αSa in the somas (s) and proximal neurites (pn) of DA neurons in three-week midbrain cultures exposed two week earlier to 0.5 μM F91. TH^+^ neurons with or without large somal aggregates (*n* = 10–11) were analyzed, separately. αSa within proximal TH^+^ neurites were quantified within a vertical projection column of a 100 μm radius centered onto individual TH^+^ cell bodies. (**d**) Z-projection of a confocal image showing an individual TH^+^ neuron (red) containing a large somal αSa (white arrowhead) and smaller neuritic aggregates in a three-week midbrain culture exposed two weeks before to 0.1 μM F91. Aggregated material appears yellow after merging red and green immunosignals. Counterstaining of cell nuclei was performed with Hoechst 33,342 (H; blue). (**e**) Side view of the DA cell body depicted in (**d**) showing that the aggregate forms a calyx-like structure at the lower part of the nucleus. Dotted lines represent virtual boundaries of the soma, the aggregate and the nucleus. (**f**) Enlargement of a neuritic segment from the TH^+^ neuron depicted in (**d**). The image shows the presence of small αSa (white arrows) along the elongated structure of the TH^+^ neurite.

To quantitatively estimate seeded αS aggregation in DA neurons, we compared two-week and three-week midbrain cultures exposed one and two weeks earlier to increasing F91 concentrations (0.01 to 0.75 μM, i.e., 0.14–10.7 μg/mL; Figure 2). Immunofluorescence analysis showed that the proportion of DA (TH^+^) cell bodies with large αS inclusions increased in a concentration- and time-dependent manner in F91-treated midbrain cultures (Figure 2a,b). Specifically, in two-week cultures analyzed one-week post-exposure to 0.1 μM fibril seeds (Figure 2a), 9.6% of the TH^+^ neurons presented large somal αSa. At the highest concentrations of fibrils, i.e., 0.5 and 0.75 μM, we estimated that the number of TH^+^ neurons with somal aggregates reached 26.5% and 41.5%, respectively. If the post-fibril exposure period was extended to two weeks, the proportion of DA cell bodies with somal aggregates rose substantially. For instance, in cultures treated with 0.05 μM F91 (Figure 2b), αS inclusions were found in 16.7% of all DA cell somas. With 0.5 and 0.75 μM of F91, the aggregation process was observable in 49.3% and 62.7% of them, respectively (Figure 2b). Importantly, the number of DA neurons was not affected under these seeding conditions, regardless of the concentration of fibrils applied to the cultures (Figure 2a,b). The presence of somal αSa was never detected in DA neurons from control cultures, irrespective of cell culture age.

Note that the seeding efficiency of F91 was quite comparable in midbrain cultures generated from Swiss mouse embryos instead of C57BL/6J mouse embryos (Figure S1). DA cell survival was also unaffected in these cultures (Figure S1).

Using 3D image analysis, we comparatively estimated the intensity of the aggregation process in cell bodies and neurites from DA neurons exposed to fibril seeds. Because of technical constraints, we analyzed only proximal neurites contained inside a vertical projection column of a 100 μm radius centered onto individual DA cell bodies. Using three-week midbrain cultures exposed two weeks earlier to 0.5 μM fibrils, we found that αSa occupied a cumulated volume of 217.3 and 24.2 μm^3^ in TH^+^ cell bodies and their proximal neurites, respectively, when considering DA neurons with large somal aggregates (Figure 2c). The corresponding values were estimated to be 0.5 and 5.6 μm^3^ when considering the subpopulation of DA neurons without large somal aggregates (Figure 2c).

The process of αS seeded aggregation is illustrated by different microphotographs in Figure 2d–f. Figure 2d is a Z-projection image of a representative individual DA (TH^+^) neuron and its neuritic network in a three-week culture exposed two weeks earlier to 0.1 μM F91 fibril seeds. The picture shows the presence of a large somal inclusion formed of filamentous-like elements that are strongly immunopositive for p-αS as well as numerous smaller aggregates along neurites originating from this neuron. Figure 2e is a 3D lateral view of the soma of the same neuron, showing that the inclusion forms a calyx-like structure at the bottom part of the nucleus. Figure 2f is a magnified image of one of the proximal TH^+^ neurites from the same DA neuron. As a whole, this is reminiscent of what we have also observed in individual mouse hippocampal cultured neurons exposed to F91 [46].

### 3.3. Seeded αS Aggregation in DA Neurons Is Not Influenced by the Degree of Maturation of the Midbrain Cultures

**Figure 3:**
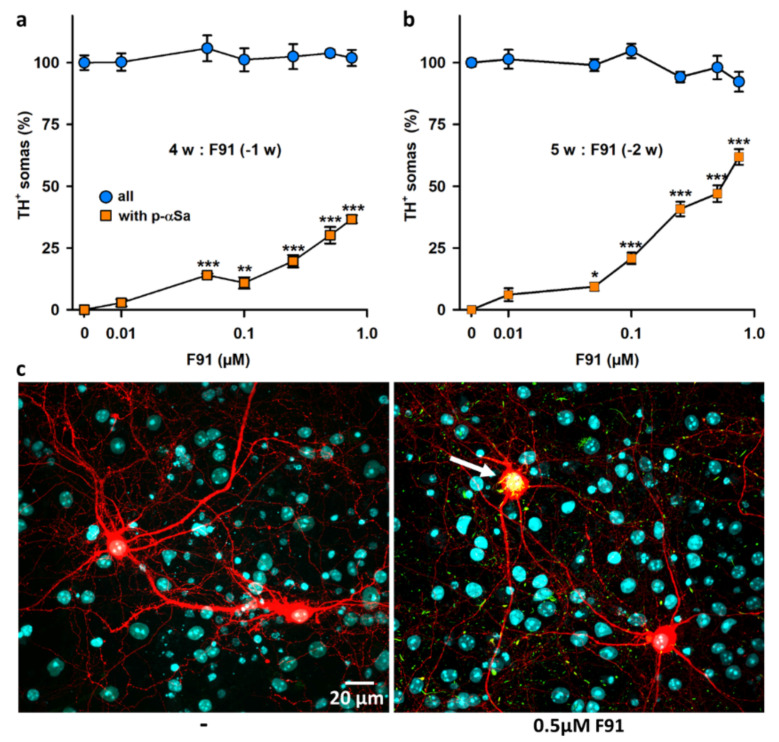
Impact of culture age on αS seeded aggregation in midbrain cultured DA neurons. (**a**) Percentage of DA (TH^+^) cell somas with αSa and survival of these neurons in four-week midbrain cultures exposed 1 week earlier (−1 w) to increasing concentrations of F91 (0.01–0.75 μM). (**b**) Same parameters as in (**a**) in five-week midbrain cultures exposed two weeks earlier (−2 w) to increasing concentrations of F91 (0.01–0.75 μM). Data in (**a**,**b**) are means ± SEM (3–8). * *p* < 0.05, ** *p* < 0.01, and *** *p* < 0.001 vs. controls. One-way ANOVA followed by Dunnett’s test. (**c**) Representative confocal images of TH^+^ neurons (red) in five-week midbrain cultures exposed or not exposed two weeks earlier to 0.5 μM F91. The large p-αS inclusion pointed out by the white arrow in one of the two DA cell bodies in F91-treated cultures appears yellow after merging the green (p-αS) and red (TH) signals. Nuclei are counterstained with Hoechst 33,342 (blue).

To determine whether cell culture age could modulate the intensity of the seeding process in DA neurons, treatments with F91 (0.01 to 0.75 μM) were initiated in three-week (instead of one-week) midbrain cultures and these cultures were processed for immunoanalysis one or two weeks later. Similar to what we reported with younger midbrain cultures, we found that the proportion of DA cell somas with inclusions increased with F91 concentrations and post-exposure time (Figure 3a,b). However, contrary to our expectations, the seeding process was not significantly enhanced in more mature DA neurons, regardless of the concentration of fibril seeds applied to the cultures. DA cell loss was also not detectable under these conditions (Figure 3a,b). These results are illustrated by microphotographs in Figure 3c.

### 3.4. Somal αSa Are Preferentially Observed in DA Neurons

**Figure 4:**
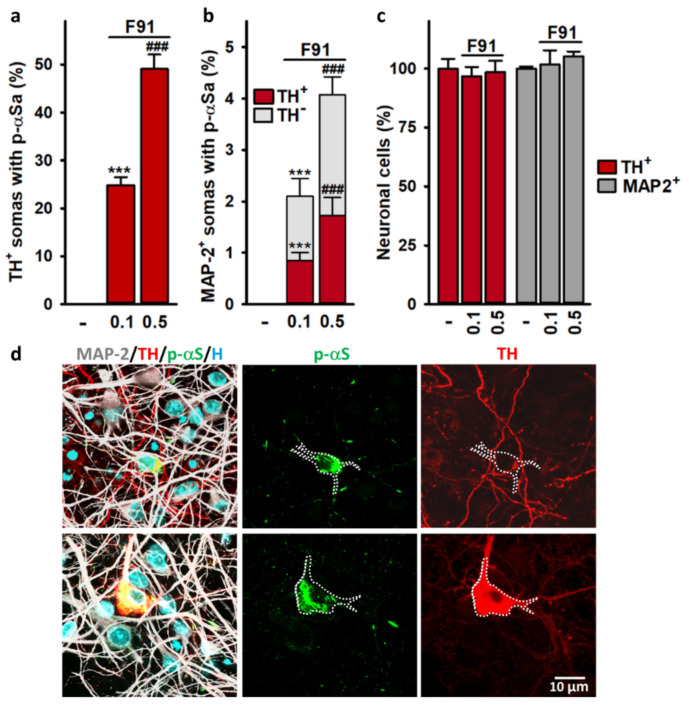
Comparative assessment of αS seeded aggregation in dopaminergic and non-dopaminergic neurons in midbrain cultures. (**a**) Number of dopaminergic (TH^+^) somas containing large αSa in three-week midbrain cultures exposed or not exposed two weeks earlier to 0.1 and 0.5 μM F91. Data are means ± SEM (*n* = 4–6). *** *p* < 0.001 vs. controls; ^###^ *p* < 0.001 vs. 0.1 μM F91. One-way ANOVA followed by SNK test. (**b**) Stacked bar graph showing the percentage of dopaminergic (TH^+^) and non-dopaminergic (TH^−^) somas with aggregates among MAP-2^+^ neurons in three-week midbrain cultures exposed two weeks earlier to 0.1 and 0.5 μM F91. Data are means ± SEM (*n* = 4–6). *** *p* < 0.001 vs. controls; ^###^ *p* < 0.001 vs. 0.1 μM F91. One-way ANOVA followed by SNK test. (**c**) Number of TH^+^ and MAP-2^+^ neurons in three-week midbrain cultures treated or not treated with 0.1 or 0.5 μM of F91 two weeks earlier. Data are means ± SEM (*n* = 4–6). (**d**) Merged and single-color images from three-week midbrain cultures exposed two weeks earlier to 0.1 μM F91 and then immunostained for MAP-2 (light grey), p-αS (green) and TH (red). Nuclear counterstaining (blue) is with Hoechst 33342. *Top panel*: Somal p-αSa in a MAP-2^+^ neuron immuno-negative for TH. *Bottom panel:* Somal p-αSa in a MAP-2^+^ neuron immunopositive for TH. In single-channel images from the top and bottom panels dotted lines indicate the virtual boundaries of MAP-2^+^ cell bodies with αS inclusions.

In a next step, we evaluated the process of αS seeded aggregation in all midbrain neurons regardless of their neurotransmitter phenotype. For this, three-week midbrain cultures exposed two weeks earlier to 0.1 and 0.5 μM F91 were processed for TH (#22941, ImmunoStar), p-αS (EP1536Y; Abcam) and MAP-2 (ab5392; Abcam) immunofluorescence analysis. In this particular experiment, large p-αS aggregates were detectable in 24.8% and 49.1% of TH^+^ somas two weeks after exposure to 0.1 and 0.5 μM F91, respectively (Figure 4a). By comparison, p-αSa were detectable in only 2.1% and 4.1% of MAP-2^+^ neuronal somas in midbrain cultures receiving the same treatments (Figure 4b). Note that these percentages were even lower when considering the number of non-dopaminergic neuronal cell bodies (MAP-2^+^/TH^−^; Figure 4b). To be precise, we estimated that about 1.3% and 2.4% of MAP-2^+^/TH^−^ somata contained αSa in cultures treated two weeks earlier with 0.1 and 0.5 μM F91, respectively. This means that the efficiency of F91 to seed αS aggregation was about 20 times greater in dopaminergic cell bodies than in the non-dopaminergic ones. Note that neither TH^+^ nor MAP-2^+^ cell numbers were significantly affected under such treatment conditions (Figure 4c). Figure 4d illustrates the fact that αS inclusions were relatively rare among MAP-2^+^ cell bodies that did not express TH.

### 3.5. Impact of DA and DA Oxidative Metabolism on αS Seeded Aggregation

**Figure 5:**
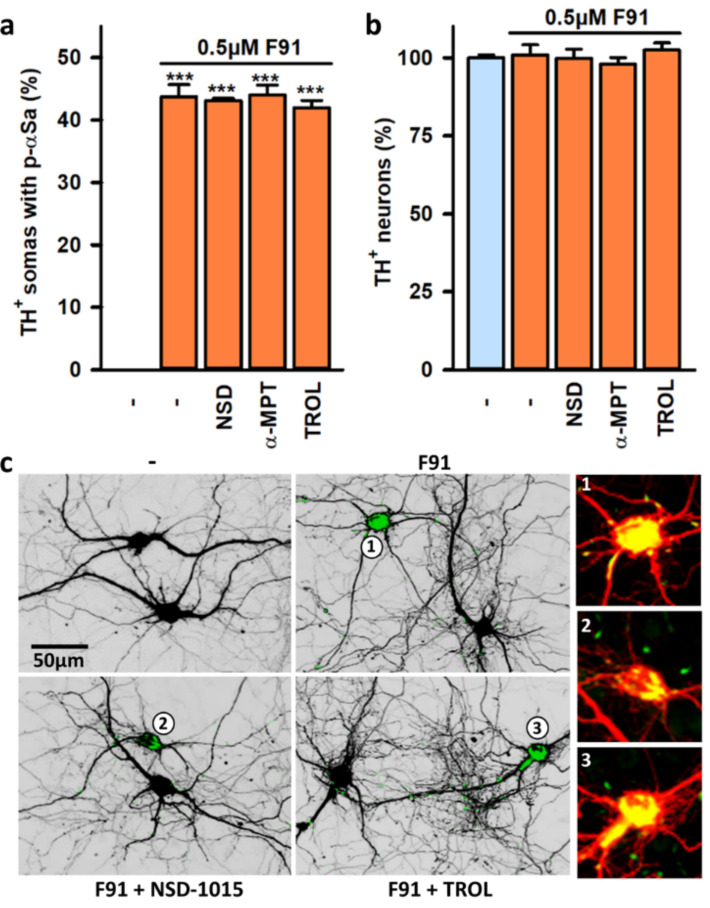
Impact of DA synthesis/catabolism on αS seeded aggregation in midbrain DA neurons in culture. (**a**) Number of TH^+^ neurons with somal αSa in three-week midbrain cultures treated or not treated two weeks before with 0.5 μM F91 in the presence or absence of the AADC inhibitor NSD-1015 (20 μM), the TH inhibitor α-MPT (50 μM) or the antioxidant Trolox-C (TROL; 10 μM). Data are means ± SEM (*n* = 4–8). *** *p* < 0.001 vs. controls. One-way ANOVA followed by Dunnett’s test. (**b**) Counts of TH^+^ neurons in cultures exposed to the same treatments as in (**a**). Data are means ± SEM (*n* = 4–8). (**c**) Z-projection confocal images showing the presence of αSa in the soma of TH^+^ neurons in midbrain cultures exposed to the same treatments as in (**a**). TH^+^ neurons are presented under an inverted black and white format and the immunosignal for p-αS corresponds to the green label. An enlarged version of the annotated TH^+^ cell bodies (red; 1–3) with large aggregates (yellow after merging red and green) is presented under a standard fluorescent format.

As αSa were preferentially observed in DA neurons, we determined whether seeding induced by F91 was possibly influenced by DA or its oxidative metabolites. To this end, we explored the extent of seeding in three week-midbrain cultures exposed two weeks earlier to 0.5 μM F91 in the presence or absence of the tyrosine hydroxylase inhibitor 3-hydroxybenzylhydrazine dihydrochloride (NSD-1015; 20 μM) or the aromatic L-amino acid decarboxylase inhibitor (AAAD) α-methyl-p-tyrosine (α-MPT; 50 μM). The concentrations of NSD-1015 and α-MPT were selected based on previous reports demonstrating their efficacy in relevant cell-based assays [47]. Treatments with these two compounds initiated 4 h prior to starting fibril exposure were renewed twice a week. Our results clearly show that neither NSD-1015 nor α-MPT reduced the proportion of DA neurons with somal αSa in these cultures (Figure 5a). Trolox-C, a vitamin E soluble analog that operates by inhibiting lipid peroxidation [48] was similarly ineffective when added to midbrain cultures exposed to F91. The survival of F91-treated DA neurons remained unaffected with all the above listed treatments (Figure 5b). The previously described quantitative data are illustrated by representative microphotographs in Figure 5c.

### 3.6. Subcellular Monitoring of αS Seeded Aggregation in DA Neurons with ^ATTO^F91

**Figure 6:**
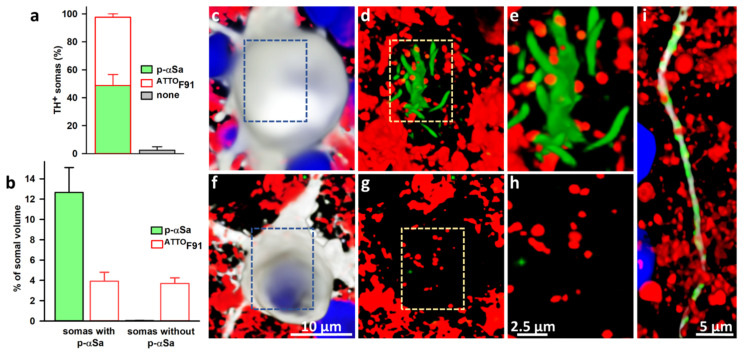
Subcellular monitoring of αS seeded aggregation in DA neurons with fluorescently labelled fibril seeds. (**a**) In three-week midbrain cultures exposed to ^ATTO^F91 (0.5 μM) two weeks earlier, fibrils are detectable in most of TH^+^ cell somas. The efficacy of ^ATTO^F91 to seed aggregation of endogenous αS in DA neurons is similar to that of unlabeled F91. (**b**) Volume occupied by p-αSa and ^ATTO^F91 in DA cell somas with or without large somal aggregates. (**c**) 3D projection of a confocal image stack showing a DA (TH^+^) cell body (light grey pseudocolor for deep red fluorescence) in a three-week midbrain culture exposed two weeks earlier to 0.5 μM ^ATTO^F91 (red). (**d**) Corresponding 3D image where the light grey channel for TH was removed to show that this soma contains small puncta of fibril seeds (red) and a large filamentous inclusion of p-αS (green). Some of the fluorescent puncta (red) seem to decorate the serpentine-like elements of the inclusion (green). (**e**) Enlarged view of the section delimited by the dotted line rectangle in (**d**). (**f**) 3D projection of a DA (TH^+^) cell body (light grey) in a midbrain culture exposed two weeks earlier to 0.5 μM ^ATTO^F91 (red). (**g**) Corresponding image without the light grey channel to show that this soma contains ^ATTO^F91 puncta but no large inclusion of p-αS. (**h**) Enlarged view of the section delimited by the dotted line rectangle in (**g**). (**i**) 3D projection image showing that ^ATTO^F91 puncta (red) are juxtaposed with elongated p-αS structures (green) along TH^+^ neurites (light grey) in a DA neuron containing a somal inclusion. Nuclear counterstaining with Hoechst-33342 (H; blue) was removed from (**d**,**e**,**g**,**h**) to allow for better visualization of somal fibril seeds.

To further characterize the process of αS seeded aggregation in DA neurons, we used F91 with an ATTO-550 fluorescent label. ^ATTO^F91 seeded the aggregation of endogenous αS to the same extent as unlabeled F91. In three-week cultures exposed two weeks earlier to 0.5 μM of ^ATTO^F91, we detected large p-αSa in 48.7% of DA cell bodies (Figure 6a). As expected, fluorescent fibril seeds were readily detectable in DA cell bodies with αSa. More unexpectedly, however, seeds were also present in almost all DA cell bodies without aggregates. Completing these observations, we found that the average volume occupied by fibril seeds was very similar in DA cell bodies with or without αS inclusions; specifically, we found that fibril seeds occupy 3.9% and 3.7% of the somal volume in DA cell bodies with and without p-αSa, respectively. As a matter of comparison, αSa occupied 12.6% of the somal volume in DA neurons with somal aggregates (Figure 6b).

Confocal images from three-week cultures exposed two weeks earlier to 0.5 μM F91 show the presence of fibril seed puncta in a DA cell body containing a large aggregate of p-αS (Figure 6c–e) and in another without any aggregates (Figure 6f–h). Fibril seed puncta were generally of small diameter (<1 μm) and were not immunopositive for p-αSa. In DA cell bodies containing p-αSa, some of the small fibril seed puncta seemed to decorate large aggregates of αS. Note that fibril seeds were also found closely juxtaposed to much smaller αSa in DA neuritic extensions (Figure 6i).

### 3.7. Impact of Mitochondrial Dysfunction on αS Seeded Aggregation

To study the potential impact of mitochondrial dysfunction on αS seeded aggregation and ensuing neurodegenerative events, we initially used treatment regimens in which F91-treated midbrain cultures were also challenged with the mitochondrial complex I inhibitor 1-methyl-4-phenylpyridinium (MPP^+^).

**Figure 7:**
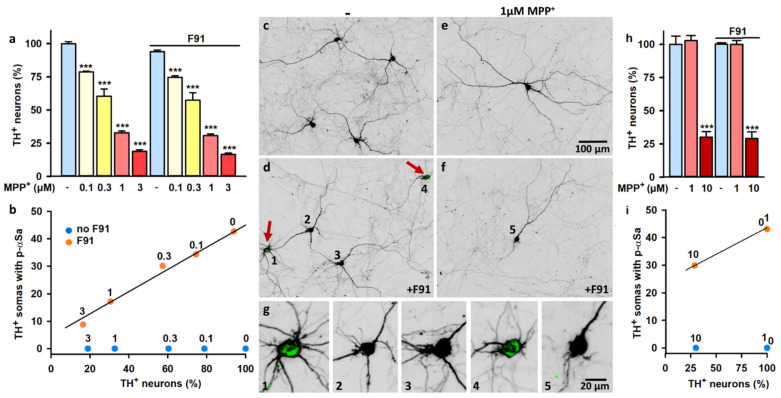
Impact of mitochondrial complex I inhibition on αS seeded aggregation in midbrain DA neurons in culture. (**a**) Survival of TH^+^ neurons in DIV 21 midbrain cultures challenged or not challenged with MPP^+^ (0.1–3 μM) between DIV 5–7 and then exposed to 0.5 μM F91 at DIV 7. Data are means ± SEM (*n* = 3–11). *** *p* < 0.001 vs. controls. One-way ANOVA followed by Dunnett’s test. (**b**) Survival of TH^+^ neurons plotted against the percentage of these neurons with somal αSa in DIV 21 midbrain cultures submitted to the same treatments as in (**a**) (*n* = 3–11). (**c**–**f**) Low magnification images from DIV 21 midbrain cultures challenged or not challenged with 1 μM MPP^+^ between DIV 5–7 and then exposed or not exposed to 0.5 μM F91 at DIV 7. Red arrows point to TH^+^ neurons with somal aggregates (green). (**g**) Enlarged version of TH^+^ somas annotated (1–5) in low-magnification microphotographs. The green signal corresponds to p-αSa. Note the absence of somal aggregates in the TH^+^ neuron surviving the MPP^+^ challenge. (**h**) Survival of TH^+^ neurons in DIV 21 midbrain cultures exposed or not exposed to 0.5 μM F91 at DIV 7 and then challenged or not challenged with MPP^+^ (1; 10 μM) between DIV 14–16. Data are means ± SEM (*n* = 4–6). *** *p* < 0.001 vs. controls. One-way ANOVA followed by Dunnett’s test. (**i**) Survival of TH^+^ neurons plotted against the percentage of these neurons with somal αSa in DIV 21 midbrain cultures submitted to the same treatments as in (**h**) (*n* = 4–6). MPP^+^ concentrations are indicated in micromolar units above circles in (**b**,**i**).

To begin with, we used DIV 21 midbrain cultures treated with 0.1–3 μM MPP^+^ between DIV 5–7 just prior to exposure to 0.5 μM F91 at DIV 7. Consistent with studies performed under quite similar conditions [49], we found that MPP^+^ alone promoted DA cell death in a concentration-dependent manner (Figure 7a), with an EC50 estimated at 0.52 μM. Importantly, we established that MPP^+^ toxic effects were not aggravated by exposure to 0.5 μM F91 (Figure 7a). Yet, in MPP^+^-treated cultures, the proportion of TH^+^ neurons with somal αSa declined progressively with increasing concentrations of the toxin (Figure 7b); on average, 42.6% of TH^+^ cell bodies contained large αSa in cultures not receiving MPP^+^, whereas this percentage dropped to 17.1% and 8.8% in cultures exposed to 1 and 3 μM of the toxin, respectively. These results are illustrated by representative photomicrographs in Figure 7c–g.

We also conducted a comparable evaluation in DIV 21 midbrain cultures initially exposed to 0.5 μM F91 at DIV 7 and then to MPP^+^ between DIV 14–16 (Figure 7h). In agreement with a previous report from Danias and colleagues [50], we found that the susceptibility of DA neurons to MPP^+^-induced neurodegeneration declined with the degree of maturation of midbrain cultures; a concentration of 10 μM MPP^+^ was required to kill about 70% of cultured DA neurons under such conditions (Figure 7h). As shown before in the previous treatment paradigm, exposure to F91 did not aggravate the loss of DA neurons induced by MPP^+^, but the percentage of DA neurons with somal aggregates was again substantially reduced (from 44.1% to 29.9%) after toxin exposure (Figure 7i). Note that the percentage of DA neurons with somal αS inclusions remained unchanged in F91-treated cultures challenged with 1 μM MPP^+^, a concentration of toxin having no impact per se on DA cell survival in the present experimental setting. Finally, we could not detect the presence of somal αSa in DA neurons solely exposed to MPP^+^, regardless of the treatment paradigm used with the toxin (Figure 7b,i).

**Figure 8:**
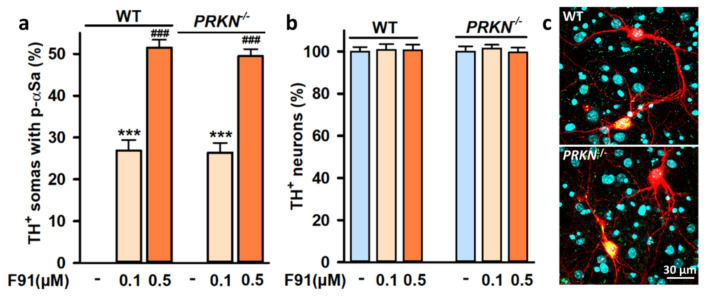
Impact of *PRKN* gene deletion on αS seeded aggregation in midbrain cultured DA neurons. (**a**) Percentage of TH^+^ neurons with somal αSa in three-week wild-type (WT) and *PRKN^−/−^* midbrain cultures exposed two weeks earlier to 0.1 or 0.5 μM F91. Data are means ± SEM (8–12). *** *p* < 0.05, vs. controls and ^###^ *p* < 0.001 vs. 0.1 μM F91. One-way ANOVA followed by SNK test. (**b**) Survival of TH^+^ neurons in the same culture conditions as in (**a**). Data are means ± SEM (8–12). (**c**) Representative confocal images from TH^+^ neurons (red) in three-week wild-type and *PRKN^-/-^* midbrain cultures exposed two weeks earlier to 0.5 μM F91. Large αSa in DA cell bodies appear yellow after merging green (p-αS) and red (TH). Hoechst-33342 nuclear counterstaining (blue).

Complementary to this, we tested whether F91-mediated seeded aggregation was possibly enhanced in midbrain cultures generated from mouse embryos harboring a deletion of the mitochondrial quality control gene *PRKN* [51]. For this purpose, we compared the intensity of αS seeded aggregation in three-week *PRKN^−/−^* and wild-type midbrain cultures exposed two weeks earlier to 0.1 or 0.5 μM F91. Microscopic examination revealed that the number of DA cell bodies with αSa was quite similar in *PRKN^−/−^* and wild-type DA neurons when considering the same concentration of fibril seeds (Figure 8a). Importantly, somal αSa were absent from *PRKN^−/−^* DA neurons not exposed to F91 seeds. Note that fibril seeds failed to promote DA cell death in *PRKN^−/−^* midbrain cultures as they did in wild-type cultures (Figure 8b). These results are illustrated by the microphotographs shown in Figure 8c.

### 3.8. DA Cell Loss Occurs When Increasing the Lag Phase after Exposure to F91

**Figure 9:**
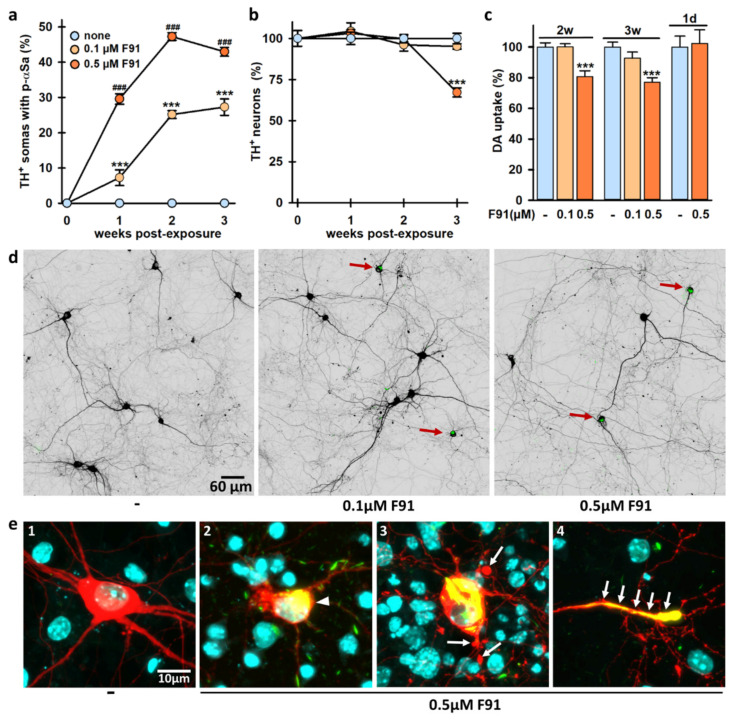
DA cell loss induced by αS seeded aggregation in midbrain cultures. (**a**) Percentage of TH^+^ neurons with somal aggregates in two-week, three-week and four-week midbrain cultures exposed one, two and three weeks earlier, respectively to 0.1 or 0.5 μM F91. Data are means ± SEM (4–9). *** *p* < 0.001, vs. controls and ^###^ *p* < 0.001 vs. 0.1 μM F91. One-way ANOVA followed by SNK test. (**b**) Survival of TH^+^ neurons in midbrain cultures exposed to same treatments as in (**a**). Data are means ± SEM (4–9). *** *p* < 0.001, vs. controls. One-way ANOVA followed by Dunnett’s test. (**c**) Tritiated-DA uptake in three-week and four-week midbrain cultures exposed two weeks and three weeks earlier, respectively, to 0.1 and 0.5 μM F91. Some of the three-week cultures were treated with 0.5 μM F91 only one day before being assayed for DA uptake. Data are means ± SEM (8–14). *** *p* < 0.001, vs. controls. One-way ANOVA followed by Dunnett’s test. (**d**) Representative low-magnification microphotographs showing TH^+^ neurons in four-week midbrain cultures exposed or not exposed three weeks earlier to 0.1 μM or 0.5 μM F91. αS inclusions (green) in TH^+^ soma are pointed out by red arrows. (**e**) Confocal images describing morphological changes affecting TH^+^ neurons in four-week midbrain cultures exposed three weeks before to 0.5 μM F91. (**1**) TH^+^ (red) neuron with an intact morphology in a control culture. (**2**) Cell body shrinkage (white arrowhead) of a TH^+^ neuron showing a large, rounded inclusion (yellow after merging green and red). (**3**) Blebs (white arrows) onto proximal neurites of a TH^+^ neuron with a large somal αS inclusion. (**4**) Distal TH^+^ neuritic extension packed with p-αS material (white arrows). Nuclei are counterstained with Hoechst-33342 (blue).

As the time elapsed after fibril exposure may be critical for unmasking αS-mediated neurodegenerative events, we monitored αS aggregation kinetics and DA cell survival up to three weeks after seeding initiation. Precisely, we performed immunoanalyses in two-week, three-week and four-week midbrain cultures treated one week, two weeks and three weeks earlier, respectively, with 0.1 and 0.5 μM of F91 (Figure 9). As expected, seeded aggregation in DA cell bodies was always more robust at the highest concentration of F91 seeds (i.e., 0.5 μM), irrespective of post-exposure time to fibrils. However, whereas seeded aggregation in DA cell bodies augmented progressively during the first two weeks post-exposure to 0.1 and 0.5 μM F91, it then either slightly decreased or plateaued at these two concentrations, respectively, three weeks post-exposure to F91 (Figure 9a). Most importantly, about 30% of TH^+^ neurons were lost in four-week midbrain cultures receiving 0.5 μM F91 three weeks earlier (Figure 9b).

Interestingly, at two weeks post-exposure to 0.5 μM (but not 0.1 μM) of F91, we also constantly detected a small reduction of about 20% of the uptake of DA (Figure 9c), an energy-dependent transport process that reflects DA cell function [34,49]. This deficit slightly progressed when the uptake was measured three weeks post-fibril exposure. Importantly, DA uptake was not reduced when the treatment with 0.5 μM F91 was performed only 24 h before carrying out the assay, i.e., in conditions where αS seeded aggregation was not yet detectable.

The photomicrographs presented in Figure 9d illustrate the loss of DA neurons observed three weeks post-exposure to 0.5 μM (but not 0.1 μM) F91. Note that under conditions leading to neurodegeneration, morphological alterations were manifest in some DA neurons with large somal inclusions (Figure 9(e2–e4)). In particular, some of these neurons were characterized by a shrinkage of their cell body, whereas others showed blebbing and fragmentation of proximal neurites. Occasionally, distal DA neurites were also found to be packed with p-αS material, making them closely resembling LNs.

### 3.9. DA Cell Loss Induced by F91 Is Preventable by Trophic Stimulation with GDNF

**Figure 10:**
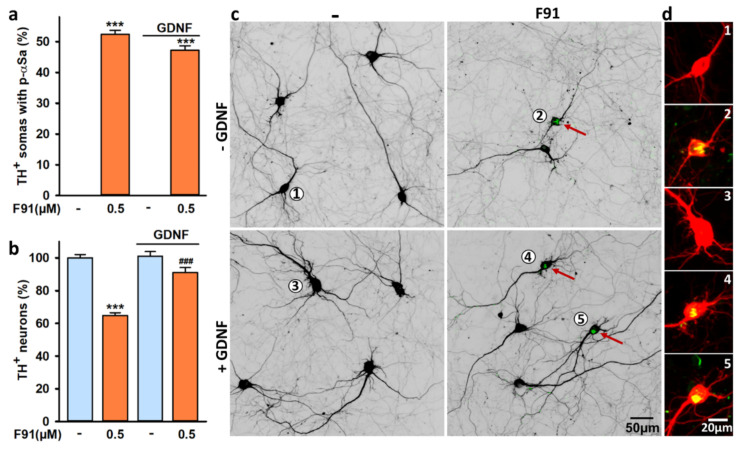
DA cell loss induced by αS seeded aggregation in midbrain cultures is preventable by GDNF. (**a**) Percentage of TH^+^ neurons with somal aggregates in four-week midbrain cultures exposed three weeks earlier to 0.5 μM F91 in the presence or the absence of 20 ng/mL GDNF. Data are means ± SEM (*n* = 4–6). *** *p* < 0.001, vs. controls. One-way ANOVA followed by SNK test. (**b**) Survival of TH^+^ neurons in midbrain cultures exposed to same treatments as before. Data are means ± SEM (*n* = 4–6). *** *p* < 0.001, vs. controls. ^###^ *p* < 0.001, vs. F91, only. One-way ANOVA followed by SNK test. (**c**) Representative microphotographs of four-week midbrain cultures exposed three weeks earlier to 0.5 μM F91 in the presence or absence of 20 ng/mL GDNF. TH^+^ neurons are presented under an inverted format and somal αSa (green) are pointed out by red arrows. (**d**) Enlarged version of annotated TH^+^ somas (**1**–**5**) shown under a standard fluorescent format. Large αSa appear yellow after merging green (p-αS) and red (TH) immunosignals.

We wished here to determine whether the trophic factor GDNF [52,53] was capable of preventing F91-induced DA cell loss. To test this possibility, we analyzed four-week midbrain cultures treated three weeks earlier with 0.5 μM F91 in the presence or absence of GDNF (Biotechne RD Systems; Abingdon, UK) added 4 h prior to initiating fibril exposure. We used a concentration of 20 ng/mL—reported previously to be optimally effective in protecting DA neurons in different cell culture settings [43,54]. Some cultures also received GDNF for 3 weeks in the absence of fibrils. Although GDNF treatment did not significantly reduce the percentage of DA neurons with αS somal inclusions (Figure 10a), it efficiently protected them from F91-induced neurodegeneration (Figure 10b). In contrast, GDNF did not enhance DA cell survival in cultures not exposed to fibril seeds. Illustration of these results is provided by the confocal images in Figure 10c,d.

## 4. Discussion

We here established a cell culture model in which midbrain DA neurons accumulate aggregates of p-αS when exposed to F91 seeds generated from recombinant human αS. The aggregation process was exquisitely specific to DA neurons in the current experimental conditions. The severity of the aggregation process hinged primarily on fibril seed concentrations and time elapsed after fibril exposure. However, three weeks after fibril exposure, αS aggregation reached a plateau phase. At this stage, DA cell death was evident in cultures exposed to optimal concentrations of fibrils. Under such conditions, DA cell loss was preceded by early deficits in DA uptake, a marker of dopaminergic cell function. DA cell loss, but not αS seeded aggregation, was preventable by trophic stimulation with GDNF, suggesting that the aggregation process induces a trophic factor deprivation-like state potentially amenable to pharmacological intervention.

### 4.1. A Large Proportion of Midbrain DA Neurons Can Potentially Develop Somal αSa upon F91 Exposure

To visualize αS inclusions in midbrain cultures exposed to F91, we initially tested three antibodies, which can identify pathogenic αS phosphorylated at Serine residue 129 [44,45]. We selected one of these p-αS-specific antibodies, namely EP1536Y, which demonstrated the best specificity and sensitivity for detecting αSa in our model system. Delic and colleagues [55] came to a similar conclusion when comparing the performances of these antibodies in rodent models of αS seeding.

Using the EP1536Y antibody, we found that aggregates developed in DA cell bodies in the form of large and rather compact filamentous-like structures generally closely apposed to the nucleus, which is in agreement with previous observations made on hippocampal cultured neurons exposed to F91 [46]. Somal aggregation in DA neurons increased as a function of F91 concentrations (0.01 to 0.75 μM) and time elapsed (1–2 weeks) after exposure to fibrils. Remarkably, two weeks after exposure to the highest concentrations of F91 (0.5–0.75 μM), a large proportion of DA cell somata contained large p-αSa, indicating that F91 seeds have a strong propensity to promote the templated aggregation of endogenous αS in these neurons. Similar results were obtained when midbrain cultures were generated with Swiss mouse embryos instead of C57BL/6J mouse embryos, suggesting that the high efficiency of F91 in seeding αS aggregation in DA neurons was not due to genetic background effects from the C57BL/6J mouse line.

Even if large p-αSa were most prominent in DA cell bodies, small p-αSa were also frequently observed along neuritic extensions from these neurons. The 3D image analysis revealed that the cumulative volume of small p-αSa in proximal dopaminergic neurites was substantial, as it represented about 11% of the volume occupied by large aggregates in dopaminergic cell bodies. In contrast, αSa were less frequently detectable in proximal neurites from DA neurons without somal aggregates, suggesting that αS aggregation proceeds in a coordinated manner in the somal and neuritic compartments of DA neurons. Finally, it is worth noting that DA cell survival was not significantly affected two weeks following exposure to the highest concentrations of F91, i.e., under conditions in which the accumulation of αSa in DA cell bodies and neurites was already substantial. This suggests that DA neurons can handle the presence of aggregated proteinaceous material relatively well—at least for a while.

### 4.2. Impact of Culture Age on αS Seeded Aggregation

As ageing is considered a primary risk factor for PD [56,57], we tested whether the use of older midbrain cultures could have an impact on αS seeded aggregation and its consequences. To model the ageing process in vitro, treatments with F91 were applied to midbrain cultures that were two weeks older than in our standard protocol, and then we processed them for analysis one or two weeks later. This was technically feasible since the use of a culture medium conditioned by astrocytes facilitates culture medium exchanges and promotes the long-term maintenance of brain neuronal cultures [36,58]. Similar to what we reported with younger cultures, the seeding process in aged cultures occurred with high efficiency in DA cell bodies. It was also strictly dependent on fibril seed concentrations and the time elapsed (i.e., one or two weeks) after fibril exposure. Yet, contrary to our expectations, the proportion of DA neurons with somal aggregates was not significantly augmented in older cultures. Furthermore, older DA neurons exposed to F91 did not demonstrate higher vulnerability to degeneration. These results should be considered with some caution, however, considering that we do not know to what extent the increased maturation of the cultures reflects biological ageing.

### 4.3. Subcellular Characterization of the Seeding Process in DA Neurons

To better characterize the seeding process in DA neurons, we exposed one-week midbrain cultures to F91 fluorescently labeled with ATTO-550 and processed them for immunofluorescence analysis two weeks later. ^ATTO^F91 were as potent as the unlabeled fibrils in generating αSa in dopaminergic cell somas and neurites, indicating that the fluorescent label did not affect the seeding propensity of F91. ^ATTO^F91 were generally detectable in the cytoplasm of DA cell bodies under the form of small (<1 μm) isolated puncta, presumably consisting of fibrillar clusters.

Interestingly, DA cell bodies contained approximately the same amount of fibril seeds regardless of whether or not they developed large p-αSa. The absence of p-αSa in DA neurons containing a large amount of fibril seeds might possibly be explained by (i) the inefficacy of the phospho-specific antibody used to detect αS inclusions that are not yet fully phosphorylated in these neurons; (ii) a limited availability of αS for conformational templating in these particular neurons, as suggested before in other model systems [29,59,60]; or (iii) the incapacity of fibril seeds to reach the cytoplasm following endocytosis [61]. Our present observation might also relate to intrinsic differences of vulnerability between SN and VTA DA neurons [62,63], two populations of midbrain DA neurons that are both present in our cultures.

Interestingly, some (but not all) αS fibril puncta appeared to decorate the outer surface of large p-αSa in DA cell bodies. This spatial proximity indicated that such puncta may provide effective templates for αS aggregation. Note that fibrillary seeds within DA cell bodies were generally not immunopositive for p-αS, indicating that seeds induced αS aggregation without being themselves phosphorylated. This corroborates previous results from Gribaudo and colleagues [64] showing that αS seeded aggregation is achievable with fibrils from a variant of αS that cannot be phosphorylated on residue Ser-129.

A 3D reconstruction of the subcellular network of DA cell bodies with p-αSa allowed us to establish that somal inclusions occupy a volume that is about three-fold that of ^ATTO^F91 in vulnerable DA neurons, thus confirming the high seeding propensity of these fibrils. We also noted that small ^ATTO^F91 puncta along dopaminergic neurites were generally juxtaposed to larger elongated aggregates of p-αS, indicating that templated aggregation does probably occur in this compartment as well.

### 4.4. αSa Are Preferentially Observed in the Soma of DA Neurons

In a next step, we evaluated the efficacy of F91 in seeding the aggregation of αS in non-dopaminergic midbrain neurons using an antibody against the pan-neuronal marker MAP-2. The occurrence of large αSa was about 20 times less frequent in the soma of non-dopaminergic neurons than in dopaminergic neurons, regardless of the concentration of fibril seeds applied to the cultures. This means that the efficiency of F91 to seed αS aggregation was globally much higher in DA neurons. This finding may clearly reflect the fact that αS pathology develops in a cell-specific manner in animal models of αS seeding [18] and in PD brains [4,65,66].

Note that the percentage of DA cell bodies developing somal αS inclusions remained unchanged when the exposure to F91 was carried out in the presence of αMPT or NSD-1015, two compounds reported be highly effective in inhibiting DA synthesis in relevant cell-based assays [47]. This indicates that the strong propensity of DA neurons to form αSa in our model system is probably not directly related to the DA content of these neurons or to oxidative stress-mediated reactions resulting from the oxidative catabolism of DA. Confirming this view, we found that the lipid peroxidation inhibitor Trolox-C was also unable to reduce the formation of αSa in DA neurons.

### 4.5. Impact of Mitochondrial Dysfunction on Seeded αS Aggregation and Its Consequences

There is some evidence of cross talk between αS pathology and mitochondrial dysfunction in PD pathogenesis [67,68,69]. Notably, it has been reported that mitochondrial complex I inhibitors can promote aggregation in cells overexpressing αS [70,71], whereas abnormal accumulation of αS was found to generate mitochondrial deficits in some experimental settings [64,72,73]. We tested here whether toxin-induced mitochondrial deficits or genetically determined dysfunction of stress-induced mitochondrial quality control mechanisms could possibly modulate αS seeded aggregation in DA neurons and unmask subsequent neurodegenerative events.

We found that DA cell death induced by the mitochondrial complex I inhibitor MPP^+^, was not aggravated by a treatment with F91, irrespective of whether MPP^+^ was added to the cultures before or during exposure to fibrils. This indicates that αS aggregation did not lead in itself to significant mitochondrial complex I deficits under the present conditions. In line with this, Burtscher and colleagues [68] failed to detect mitochondrial respiration deficits in a seeding-based mouse model of αS aggregation despite pronounced αS pathology. Incidentally, we observed that lower survival rates correlated with less frequent somal aggregates in the remaining DA neurons. This suggests that DA neurons with αSa were intrinsically more sensitive to MPP^+^-mediated insults. However, considering that MPP^+^’s efficacy in killing DA neurons was not aggravated by F91, one may assume that the vulnerability of DA neurons to mitochondrial complex I inhibition and degeneration was not simply related to the presence of aggregates but also to the potential of these neurons to generate them. Studies performed on other neuronal cell types [29,59] indicate that this capacity might correlate with αS abundance. The idea that DA neuron vulnerability might be linked to αS abundance is also supported by post-mortem studies showing that αS gene expression is reduced in SN DA neurons surviving neurodegeneration in sporadic PD [74,75,76].

Then, to further investigate a possible interaction between mitochondrial dysfunction and αS aggregation, we studied whether loss-of-function of *PRKN*—a PD-associated gene encoding Parkin [77], an E3 ubiquitin ligase that contributes to mitochondrial quality control and turnover [51]—could modify αS seeded aggregation in DA neurons. Indeed, knockout of *PRKN* was reported to stimulate αS phosphorylation in SN DA neurons transduced with Adeno-Associated Virus-αS [78]. In the present setting, a loss-of-function mutation of *PRKN* did not enhance αS seeded aggregation in DA neurons, nor did it promote DA cell death in the presence of fibril seeds, indicating that defects in mitophagy and other Parkin functions do not exacerbate αS seeded aggregation and its consequences.

Finally, we failed to detect αSa in DA neurons from MPP^+^-treated cultures and *PRKN^−/−^* cultures not exposed to fibril seeds, which indicates that mitochondrial deficits were not sufficient, as such, to initiate αS-related pathological events. Consistent with these observations, Halliday and colleagues [79] did not observe LB-like aggregates in the SN of monkeys exposed to MPTP, the biological precursor to MPP^+^, and p-αS aggregates were not detected in SN DA neurons from *PRKN*^−/−^ mice [78].

### 4.6. Factors Governing αS-Dependent Neurodegeneration in F91-Treated Midbrain Cultures

As αS-dependent neurodegenerative events may develop when neuronal insults reach a critical threshold, we performed experiments in which the time lag after fibril exposure was extended by up to three weeks. Contrary to our expectations, we found that the number of DA cell bodies with p-αS aggregates was only marginally increased or even slightly decreased in four-week midbrain cultures exposed three weeks before to 0.1 or 0.5 μM of F91, respectively, suggesting that the aggregation process reached a plateau phase at this stage. At this time point, we found that the number of TH^+^ neurons was reduced by about 30% in midbrain cultures exposed to 0.5 μM (but not 0.1 μM) F91, indicating that neurodegeneration was ongoing under such conditions. This probably explains why signs of degeneration were observable in a significant number of TH^+^ neurons containing large αSa in these cultures. In particular, some of these neurons exhibited a drastic reduction of their somal volume, whereas others developed blebs onto proximal neurites. Remarkably, portions of distal TH^+^ neurites were also occasionally packed with aggregated material, making them resemble LNs in PD brains [80].

These data are also in agreement with a number of studies showing that αS aggregation results ultimately in neurodegeneration [81,82], but in contradiction with the idea that proteinaceous inclusions exert a cytoprotective function for neuronal cells that generate them [83,84,85]. Most importantly, our findings also indicate that the time elapsed after fibril exposure and fibril concentrations is a key factor controlling αS-dependent neurodegeneration in our model system.

Most interestingly, two weeks following exposure to 0.5 μM of fibrils (i.e., in conditions where DA cell loss has not yet occurred), we observed a small but constant reduction of about 20% in the uptake of DA—an energy-dependent transport process that reflects the somato-dendritic and synaptic functions of DA neurons [34]. DA uptake remained unaffected upon acute exposure to fibril seeds, i.e., in conditions where αS seeded aggregation has not yet progressed significantly. This indicates that the DA uptake deficits observed two weeks post-seeding are probably due to the progression of the aggregation process. This result is also coherent with reports showing that neurotransmission defects occur at an early stage during the course of αS inclusion formation [86,87]. Overall, these data suggest that αS-mediated neurodegeneration of DA neurons is a complex process in which the growth of αS aggregates causes neurotransmission defects that precede neuronal cell death.

### 4.7. FαS Aggregation-Dependent DA Cell Loss Is Preventable by Trophic Stimulation with GDNF

GDNF is a trophic factor with potent neuroprotective and neurorestorative properties for DA neurons in experimental settings that model PD neurodegeneration [40,50]. Here, we tested the possibility that GDNF could prevent DA cell loss in experimental conditions where αS aggregation leads to DA cell neurodegeneration. Although GDNF had no impact on DA cell survival in control culture conditions, it protected DA neurons from F91-induced neurodegeneration. Based on these results, we may assume that αS aggregation reproduced a state of trophic factor deprivation, ultimately resulting in DA cell death. It remains to be demonstrated whether the rescue of F91-treated DA neurons by GDNF requires the activation of the canonical GDNF receptor Ret and that of PI3K/Akt and RAS/MAPK effector cascades known to be involved in the survival-promoting effects of this factor [53]. Note, however, that GDNF did not significantly reduce αS aggregation in F91-treated DA neurons, leading us to assume that GDNF operated by inhibiting a death effector mechanism that is downstream of the process of αS aggregation itself. These results are only in partial agreement with another study showing that GDNF has the capacity to prevent both αS aggregation and ensuing neurodegenerative events [88]. The reasons for such a discrepancy are not immediately clear.

## 5. Conclusions

To conclude, we have established an in vitro system of mouse midbrain cultures for modeling αS-dependent neurodegeneration in PD. We showed that αS aggregation induced by fibril seeds occurs preferentially in a subpopulation of DA neurons for a reason that is not directly related to DA metabolism. Besides this, we demonstrated that αS aggregation results in DA cell death, but only when cultures are maintained for an extended period after exposure to fibril seeds. Interestingly, DA cell neurodegeneration was preventable with the trophic factor GDNF, indicating that αS aggregation may reproduce a state of trophic factor deprivation that is amenable to pharmacological intervention. Although we are well aware that this model system cannot fully recapitulate the complex and multifaceted nature of aggregation and neurodegeneration in PD, we suggest that it may be successfully employed for addressing specific PD pathophysiological mechanisms and testing molecules of therapeutic interest.

## Figures and Tables

**Table 1:** List and details of primary antibodies.

Primary Antibodies	Host	Working Dilution	Source	Identifier
anti-p-S129 αS [EP1536Y]	Rabbit	1:2500	Abcam	ab51253
anti-p-S129 αS [P-Syn#64]	Mouse	1:50,000	Wako	W1W015-25191
anti-p-S129 αS [P-syn/81A]	Mouse	1:1000	Abcam	ab184674
anti-TH [LNC1]	Mouse	1:2500	Immunostar	22941
anti-TH	Chicken	1:1000	Abcam	ab76442
anti-MAP-2	Chicken	1:500	Abcam	ab5392

## Data Availability

The datasets generated during the current study are available from the corresponding author upon reasonable request.

## Supplementary Materials

The following supporting information can be downloaded at: https://www.mdpi.com/article/10.3390/cells11101640/s1, Figure S1: Impact of mouse lineage on αS seeded aggregation in midbrain DA neurons in culture; Table S1: List and details of secondary antibodies.
