# Supplementary material for: Modelling α-Synuclein Aggregation and Neurodegeneration with Fibril Seeds in Primary Cultures of Mouse Dopaminergic Neurons
Source: Cells. 2022 May 13;11(10):1640. doi: 10.3390/cells11101640 (PMC9139621; doi:10.3390/cells11101640)
Supplement: Supplementary file 1 [file cells-11-01640-s001.zip › cells-1689482-supplementary.pdf]

Supplemental Material and Methods

Table S1: List and details of secondary antibodies

Secondary antibodies*	Host	Working dilution	Cat n°
Anti-rabbit IgG (H+L), Alexa Fluor 488	Goat	1:1000	A-11008
Anti-rabbit IgG (H+L), Alexa Fluor 647	Goat	1:300	A-21245
Anti-mouse IgG (H+L), Alexa Fluor 555	Goat	1:300	A-21422
Anti-mouse IgG (H+L), Alexa Fluor 647	Goat	1:300	A-21235
Anti-chicken IgY (H+L), Alexa Fluor 555	Goat	1:300	A-32932
Anti-chicken IgY (H+L), Alexa Fluor 647	Goat	1:300	A-32933

* *Thermo Fisher Scientific*

Supplementary results (Figure S1)

The genetic background of C57BL/6J mice may possibly influence the response of dopamine (DA) neurons to fibril seeds. Thus, we compared the impact of F91 seeds in midbrain cultures generated with either C57BL/6J or Swiss mouse embryos. Specifically, we estimated the percentages of DA cell somas with large αSa in three-week cultures exposed two weeks earlier to 0.1 or 0.5 μM of F91. Our data revealed that the percentage of DA neurons with large somal αS inclusions was quite similar in the two types of cultures, at a same concentration of fibrils (Figure S1a). DA cell survival remained also unchanged in F91-treated midbrain cultures generated from Swiss mouse embryos, regardless of the concentration of fibrils used to seed αS aggregation (Figure S1b). These results are illustrated by microphotographs in figure S1c.

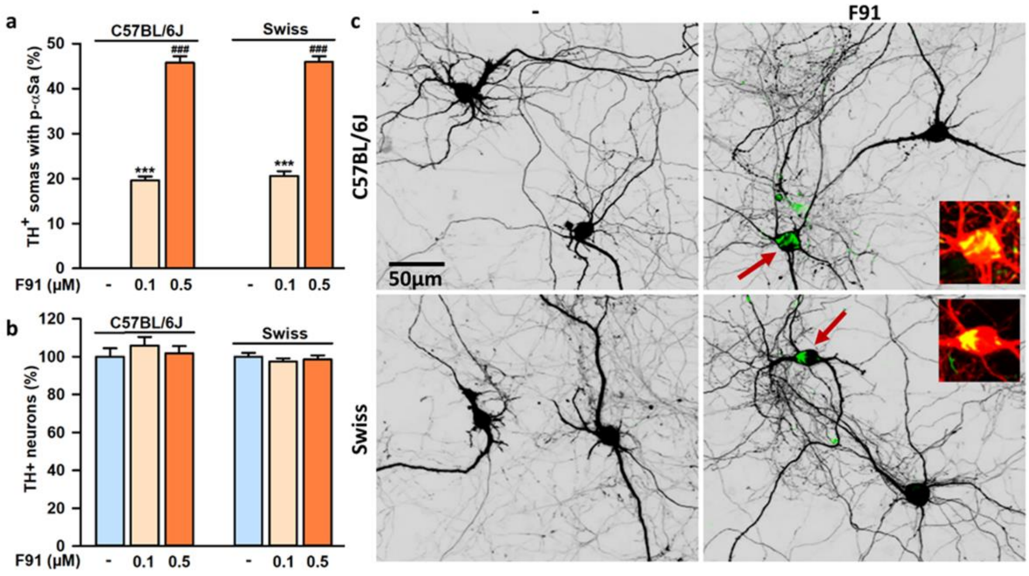

Figure S1: Impact of mouse lineage on αS seeded aggregation in midbrain DA neurons in culture. **(a)** Number of TH^+ neurons with somal aggregates in three-week C57BL/6J and Swiss mouse midbrain cultures exposed two weeks earlier to 0.1 or 0.5 μM F91. Data are means ± SEM (n=5-16). ***p<0.001 *vs.* controls and ###p<0.001 *vs.* 0.1 μM F91. One-way ANOVA followed by SNK test. **(b)** Survival of TH^+ neurons in the same culture conditions as in **(a)** (n=5-16). **(c)** Representative microphotographs of TH^+ neurons in three-week C57BL/6J and Swiss mouse midbrain cultures exposed 2 weeks earlier to 0.5 μM F91. The immunosignal for p-αS corresponds to the green label and TH^+ neurons are presented under an inverted black and white format. Red arrows point to DA cell bodies with somal αSa. An enlarged version of TH^+ somas (red) with p-αSa (yellow after merging red and green) is shown in the inserts under a standard fluorescent format.
